# Immune Modulating Brevetoxins: Monocyte Cytotoxicity, Apoptosis, and Activation of M1/M2 Response Elements Is Dependent on Reactive Groups

**DOI:** 10.3390/md20040233

**Published:** 2022-03-29

**Authors:** Jennifer R. McCall, Kathryn T. Sausman, Devon M. Keeler, Ariel P. Brown, Stephanie L. Turrise

**Affiliations:** 1 School of Nursing, College of Health and Human Services, University of North Carolina Wilmington, Wilmington, NC 28403, USA; sausmank@uncw.edu (K.T.S.); pottera@uncw.edu (A.P.B.); turrises@uncw.edu (S.L.T.); 2 SeaTox Research Inc., Wilmington, NC 28409, USA; dkeeler@seatoxresearch.com

**Keywords:** brevetoxin, inflammation, monocyte, T cell, cytotoxicity, apoptosis, stroke

## Abstract

Brevetoxins are a suite of marine neurotoxins that activate voltage-gated sodium channels (VGSCs) in cell membranes, with toxicity occurring from persistent activation of the channel at high doses. Lower doses, in contrast, have been shown to elicit neuroregeneration. Brevetoxins have thus been proposed as a novel treatment for patients after stroke, when neuron regrowth and repair is critical to recovery. However, findings from environmental exposures indicate that brevetoxins may cause inflammation, thus, there is concern for brevetoxins as a stroke therapy given the potential for neuroinflammation. In this study, we examined the inflammatory properties of several brevetoxin analogs, including those that do and do not bind strongly to VGSCs, as binding has classically indicated toxicity. We found that several analogs are toxic to monocytes, while others are not, and the degree of toxicity is not directly related to VGSC binding. Rather, results indicate that brevetoxins containing aldehyde groups were more likely to cause immunotoxicity, regardless of binding affinity to the VGSC. Our results demonstrate that different brevetoxin family members can elicit a spectrum of apoptosis and necrosis by multiple possible mechanisms of action in monocytes. As such, care should be taken in treating “brevetoxins” as a uniform group, particularly in stroke therapy research.

## 1. Introduction

Brevetoxins (PbTxs) are a family of lipid-soluble polyether neurotoxins produced by the marine dinoflagellate *Karenia brevis,* which bind to receptor site 5 on voltage-gated sodium ion channels (VGSCs) in cell membranes [1]. The binding to VGSCs causes the sodium channels to open at normal resting potentials, resulting in a sodium influx into the cell. Brevetoxin binding causes a shift of the activation potential to more negative values, increases the mean channel open time, and inhibits channel inactivation [2,3,4]. Brevetoxin activation via VGSCs leads to the elevation of intracellular calcium concentration [5], which in turn mediates many cellular effects. Brevetoxins have also been shown to upregulate N-methyl-d-aspartate (NMDA) ionotropic glutamate receptor currents [6], which is an important receptor for controlling synaptic plasticity and is essential for learning and memory. The mechanism for upregulation is likely due to intracellular sodium ions, rather than a direct NMDA receptor effect [7]. However, PbTx-2 treatment of cerebrocortical neurons resulted in NMDA receptor-mediated calcium influx [8], in addition to increased neurite outgrowth, dendritic arborization, and synaptogenesis [9]. Taken together, these results and others showing the activation of downstream calcium response elements [9] have led to the proposition that brevetoxins may be a good treatment for neuroregeneration and recovery following ischemic stroke. In fact, PbTx-2 treatment in vivo in an animal model of stroke led to increased dendritic arborization, synapse density, and improvement of motor recovery [6], further leading credence to brevetoxins as a novel treatment for stroke.

The family of brevetoxins is most notorious for the toxic effects precipitated during harmful algal blooms of *K. brevis,* and subsequent presentation of neurotoxic shellfish poisoning when contaminated shellfish are consumed by humans. However, studies focusing on environmental exposures and consequences have also shown that brevetoxins can elicit a myriad of inflammatory effects. Brevetoxins aerosolized by wind and wave action can cause eye irritation and acute respiratory irritation of both the upper and lower respiratory tracts, including nasal congestion, throat irritation, cough, chest tightness, wheezing, and shortness of breath [10]. Airway exposure to brevetoxins, from either the environment or in a lab, results in the recruitment of inflammatory cells to the respiratory tract [11,12]. Lymphocyte infiltration and inflammatory damage of the cerebellar meninges in manatees suggests that brevetoxins can become systemic, despite localized route of entry [13]. Recruitment of immune cells to the site of ischemic stroke and further activation from brevetoxin treatment would be counterintuitive to the use of brevetoxins as a potential therapeutic.

Brevetoxins have been found to increase the activation of immune cells, including oxidative responses [14,15], phagocytosis [16], and inflammatory cytokine production [16,17]. However, activation responses have shown mixed results, and some of the conflicting responses could be attributed to the different analogs of “brevetoxin” used in studies. For example, PbTx-2 (0.56 µM) caused an increase in phagocytic activity [16], whereas PbTx-3 (0.5 and 1 µM) did not [15]. Similarly, PbTx-2 (0.56 µM) increased the secretion of pro-inflammatory TNF from macrophages [16], whereas another study found that PbTx-3 (1 µM) did not induce any change in inflammatory mediator expression [18]. While some studies have still found inflammatory effects from PbTx-3, and several others found potentiating effects due to PbTx-3 and other inflammatory or infectious stimuli, these conflicting results are still quite curious considering that PbTx-2 and PbTx-3 bind with similar affinity to the VGSC receptor [19]. 

Two diverging fields of evidence exist: that brevetoxins may be a potential novel therapy following ischemic stroke, due to their neuroregenerative properties, and that brevetoxins are potent inflammatory agents that cause inflammatory cell recruitment and infiltration, as well as inflammatory activation and mediator release. While both may be true, dependent on model species studied, toxin dose, and timing (chronic vs. acute exposure), there still exists a concerning question as to whether brevetoxins would be a potential stroke therapy if they elicit inflammation, which would be counterintuitive to stroke recovery. Further complicating the field is that brevetoxins, as a family, have been treated equally, with PbTx-2 and PbTx-3 being considered interchangeable in terms of toxicity and therapeutic studies, when they are in fact two different toxins. As such, it was our intent to study the potential inflammatory effects of multiple analogs in the family of brevetoxins, including those that bind well to VGSCs, as well as those that do not, to further the understanding of brevetoxin inflammation in the ultimate hopes of clarifying the use of brevetoxins as a novel stroke therapy.

## 2. Results

### 2.1. Brevetoxin Analog Structures

**Figure 1:**
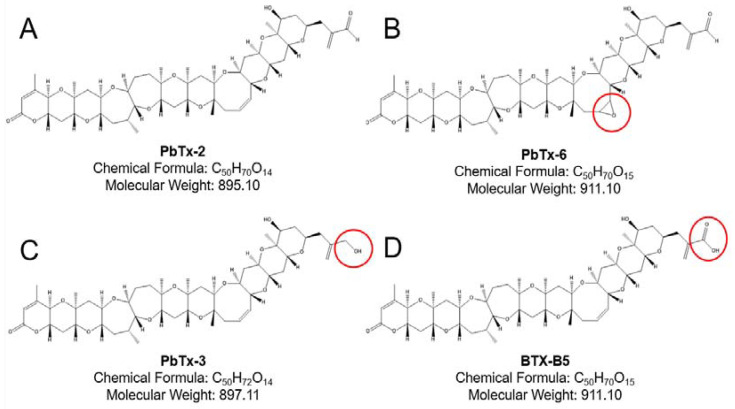
Type B brevetoxin structures. The parent type B brevetoxin PbTx-2, with the aldehyde end group, is shown in Panel **A**. Panel **B** shows PbTx-6 with the epoxide group on the H-ring of the polyether backbone. Panel **C** shows a main metabolite PbTx-3, in which the aldehyde of PbTx-2 has been reduced to an alcohol. Panel **D** shows another metabolite, BTX-B5, which differs from PbTx-2 and PbTx-3 by a carboxylic acid functional group. Red circles on Panels **B**–**D** illustrate the key structural differences from the parent PbTx-2.

The brevetoxins are a suite of cyclic polyethers based on either the type A (e.g., PbTx-1) backbone or type B (e.g., PbTx-2) backbone. Several derivatives and metabolites exist from each backbone, which are found in the culture of K. brevis, the environment during blooms, and in contaminated shellfish. The unsaturated aldehyde K-ring side chain of PbTx-2 is rapidly metabolized to various analogs [20]. Our study focused on the type B backbone analogs shown in Figure 1, as those are the proposed treatments for stroke.

### 2.2. Brevetoxin Analog Immunotoxicity Does Not Align with VGSC Receptor Binding

**Figure 2:**
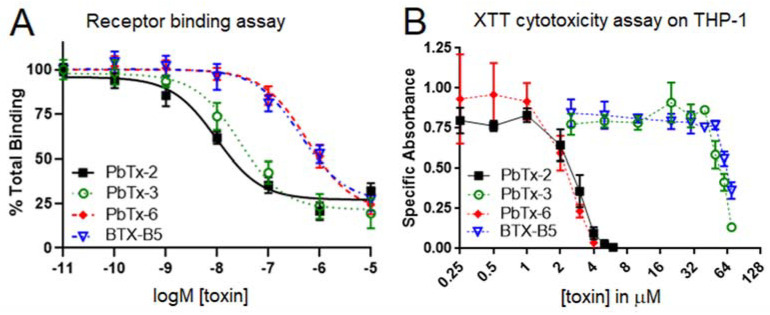
Comparison of VGSC binding vs. THP-1 cytotoxicity for the four brevetoxin B analogs. A receptor binding assay was used to measure binding of brevetoxins to the VGSC receptor (Panel **A**), whereas an XTT assay was used to measure cytotoxicity of the brevetoxins to THP-1 monocytes (Panel **B**). Black squares indicate PbTx-2, green circles indicate PbTx-3, red diamonds indicate PbTx-6, and blue triangles indicate BTX-B5. Data are shown as mean +/− standard deviation for each concentration (*n* = 3 for each assay).

To date, the major paradigm has been that brevetoxins elicit toxicity and all corresponding biological effects via binding to the VGSC. Previous studies have consistently found that tighter binding of various toxins to the VGSC positively correlates with toxicity [21,22,23], so much so that receptor binding assays have been approved by the Interstate Shellfish Sanitation Conference as a laboratory method reference for screening food for safety/toxicity from other marine toxins [24]. We tested the four analogs in Figure 1 on the receptor binding assay for VGSCs. As shown in Figure 2A, PbTx-2 and PbTx-3 showed similar binding to the receptor. This finding is consistent with what has previously been found by our lab [19,25]. In addition, PbTx-6 and BTX-B5 bound less strongly to the VGSC receptor and in a similar fashion to each other (Figure 2A). As shown in Table 1, calculations of EC_50_ values yielded significant differences between high bind vs. low bind clusters (i.e., PbTx-2/PbTx-3 vs. PbTx-6/BTX-B5) but not within binding clusters (i.e., PbTx-2 vs. PbTx-3 or PbTx-6 vs. BTX-B5). Lower EC_50_ values indicate higher affinity for the VGSC receptor. 

Our next goal was to align toxicity with binding using the XTT cytotoxicity assay on the human monocyte cell line THP-1 with the four brevetoxin analogs. However, upon analysis, a new clustering trend was observed. As seen in Figure 2B, analogs with the aldehyde functional group (i.e., PbTx-2 and PbTx-6) demonstrated high toxicity as compared to analogs without an aldehyde functional group (i.e., PbTx-3 and BTX-B5). Table 1 shows the corresponding EC_50_s for these toxins, where lower EC_50_ values indicate higher toxicity. Our results indicate that PbTx-3 bound similarly to the VGSC receptor as PbTx-2, with a roughly three-fold higher EC_50_, but was much less toxic to THP-1 monocytes, with an approximately 18-fold higher EC_50_. 

### 2.3. Inflammatory Activation of THP-1 Monocytes by Brevetoxins

**Figure 3:**
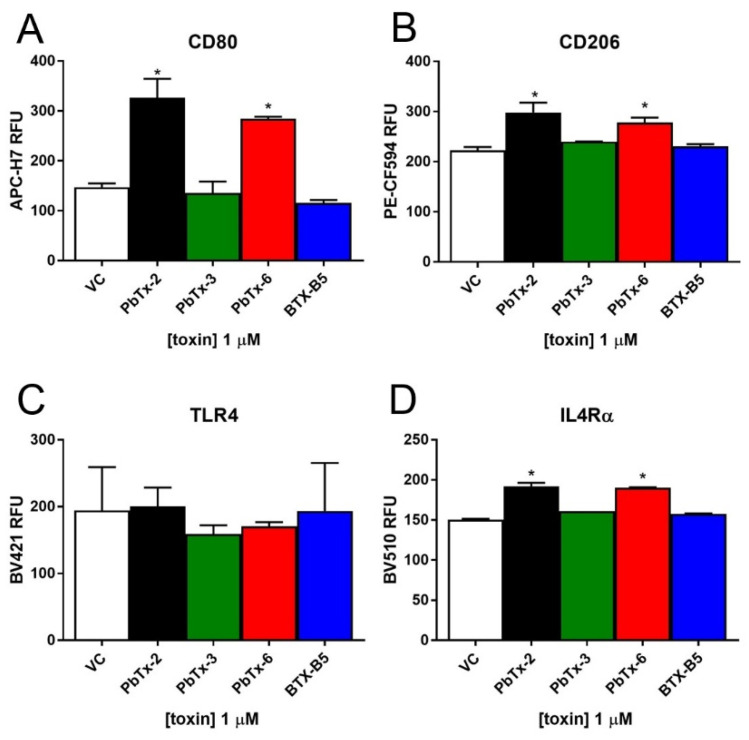
THP-1 monocyte cell-surface expression of key inflammatory response elements. THP-1 monocytes were treated with the four brevetoxin analogs and assessed for cell-surface expression of CD80 (Panel **A**), CD206 (Panel **B**), TLR4 (Panel **C**), and IL4Ra (Panel **D**) by flow cytometry. Data is presented as mean ± standard deviation (*n* = 3), with * indicating statistically significant difference from vehicle control (VC) (*p* < 0.05).

To investigate the potential cellular effects of brevetoxins, we treated THP-1 monocytes with the four analogs and examined the cell-surface expression of key inflammatory markers, as well as the inflammatory cytokine secretion. We chose a consistent dose of 1 µM, as that dose was nontoxic for all four analogs but also elicited some degree of VGSC binding. As shown in Figure 3, the aldehyde-containing brevetoxins (PbTx-2 and PbTx-6) elicited significant increases in THP-1 cell surface expression of Cluster of Differentiation (CD) 80 (Figure 3A). CD80 is an important molecule in coordinating inflammatory responses and activating T cells [26,27]. It is also a sign of M1 or the classical activation of monocytes, which typically results in a proinflammatory response. PbTx-2 yielded a 122% increase in CD80 expression over vehicle control, while PbTx-6 yielded a 93% increase in CD80 over vehicle control. To a significant yet lesser extent, aldehyde-containing brevetoxins (PbTx-2 and PbTx-6) also elicited increases in M2/alternative activation markers CD206 and Interleukin 4 receptor alpha (IL4Rα), but they did not cause changes in cell-surface expression of the lipopolysaccharide (LPS) receptor toll-like receptor (TLR) 4 (Figure 3B–D). CD206 is a mannose receptor that is important in phagocytic responses of monocytes, and IL4Ra is a receptor for the IL-4 cytokine signaling molecule that results in the alternative activation of monocytes, polarized to an M2-like or anti-inflammatory response. Both CD206 and IL4Ra are involved in resolution of inflammation [26,28]. Treatment with the other brevetoxin analogs (i.e., PbTx-3 and BTX-B5) that did not have aldehyde groups did not result in activation of any monocyte response elements measured (Figure 3). We also examined TNFα, IL-6, and IL-1β cytokine secretion following exposure to all four analogs, but none of the brevetoxins elicited measurable responses (data not shown).

### 2.4. Apoptosis and Necrosis from Brevetoxin-Treated THP-1 Monocytes

**Figure 4:**
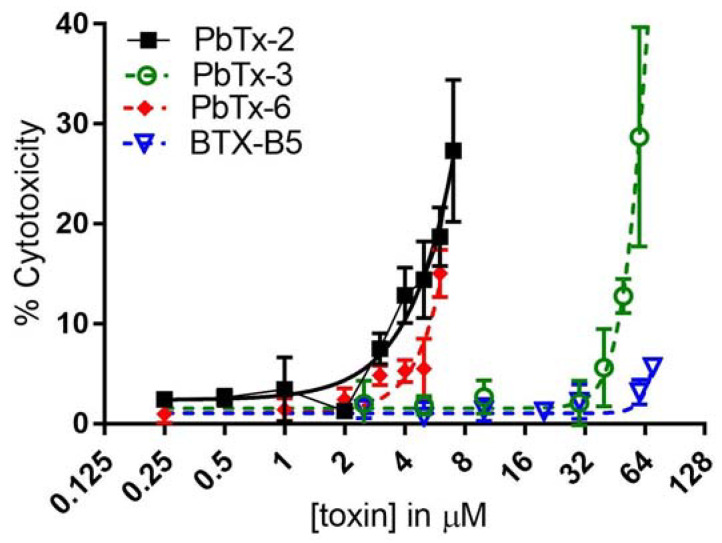
LDH release from THP-1 cells. Cells were treated with the four brevetoxin analogs at increasing doses and then assessed for LDH release in the culture supernatant. Black squares indicate PbTx-2, green circles indicate PbTx-3, red diamonds indicate PbTx-6, and blue triangles indicate BTX-B5. Data are shown as mean ± standard deviation for each concentration (*n* = three for each assay).

Lactate Dehydrogenase (LDH) is a cytosolic enzyme that is released from cells when the cell membrane is damaged during the process of cell death. LDH release from monocytes can also be an indicator of necrosis or even pyroptosis, which is programmed pro-inflammatory cell death. Necrosis is differentiated from apoptosis, where the former causes loss of membrane integrity, water influx, and lysis, and the latter results in cell shrinkage and (intact) membrane blebbing (as summarized in [29]). All brevotoxin analogs caused the release of LDH from monocytes (Figure 4). Importantly, the effect was clustered as it was for the XTT cytotoxicity, with the aldehyde-containing PbTx-2 and PbTx-6 brevetoxins, resulting in significantly higher LDH release at 4 µM and 6 µM, respectively. PbTx-3 did not cause significantly higher LDH release over baseline until 50 µM. BTX-B5 did not yield significant increases in LDH at the highest dose tested (70 µM). It may be that BTX-B5 was not tested at a high enough dose to elicit significantly higher LDH release, as indicated by the slight but not quite significant increase at 70 µM. Alternatively, the LDH release and corresponding cytotoxicity could be a combination of VGSC binding (for which PbTx-3 binds well but BTX-B5 does not) and another mechanism related to the aldehyde functional groups on PbTx-2 and PbTx-6. Studies are ongoing in our lab to elucidate these precise mechanisms of action.

**Figure 5:**
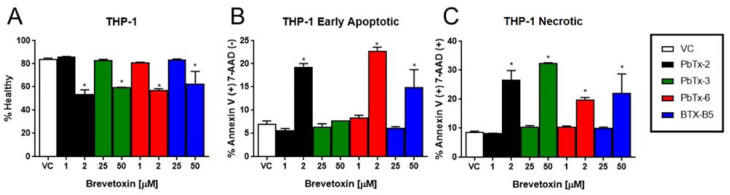
THP-1 monocytes undergo apoptosis and necrosis when exposed to brevetoxins. THP-1 cells were treated with PbTx-2, PbTx-3, PbTx-6, or BTX-B5 at approximate EC_50_ doses (2µM or 50 µM) or half EC_50_ doses (1 µM or 25 µM). Panel **A** shows the proportion of healthy cells. Panel **B** shows cells that were positive for annexin V but not 7-AAD (i.e., early apopototic), and Panel **C** shows cells that were positive for both Annexin V and 7-AAD (i.e., late apoptotic or necrotic). Data is presented as mean ± standard deviation, with * indicating a statistically significant difference from vehicle control (VC).

**Figure 6:**
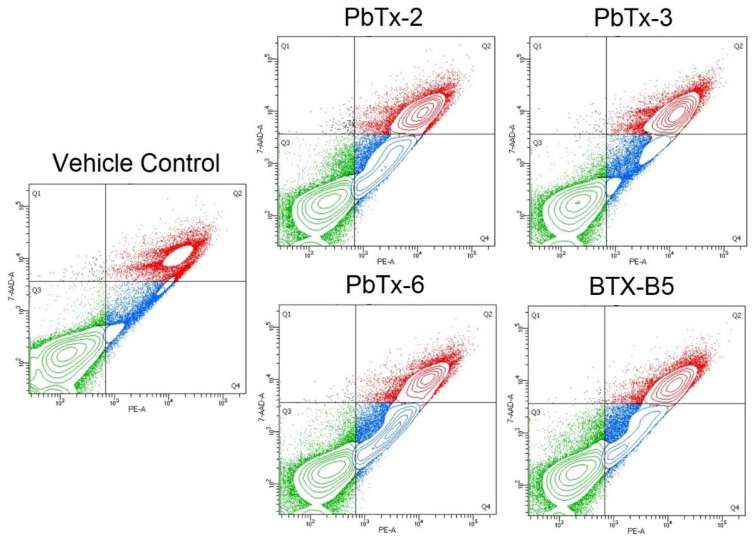
Gating strategy for determining apoptosis and necrosis for toxin treatment on THP-1 monocytes. Green quadrant (Q3) is healthy cells (7-AAD low/PE-annexin V low), blue quadrant (Q4) is early apoptotic cells (7-AAD low/PE-annexin V high), and red quadrant (Q2) is late apoptotic/necrotic cells (7-AAD high/PE-annexin V high).

In order to examine the mechanisms of cell death in a cell-by-cell basis (rather than in aggregate, as the LDH assay measures), THP-1 monocytes were treated with brevetoxins and stained for flow cytometry using an annexin V antibody and 7-aminoactinomycin D (7-AAD), which intercalates into double stranded DNA. Cells that are positive for Annexin V only are undergoing early apoptosis, as Annexin V binds to phosphatidylserine, an intracellular membrane protein that flips to external when apoptosis occurs. Those cells that are double positive for both annexin V and 7-AAD are undergoing late apoptosis, when membrane integrity is compromised, or necrosis because loss of membrane integrity allows for 7-ADD and annexin V (and the annexin V antibody) to enter the permeable cell and bind to their targets. As seen in Figure 5, all toxins exhibited reduced viability at the corresponding approximate EC_50_ dose for each respective toxin. All toxins increased late apoptosis/necrosis at the EC_50_ doses. However, PbTx-3 did not elicit a significant increase in early apoptosis, whereas the other three toxins did (Figure 5). Representative examples and the gating strategy for determining apoptosis vs. necrosis are shown in Figure 6. 

**Figure 7:**
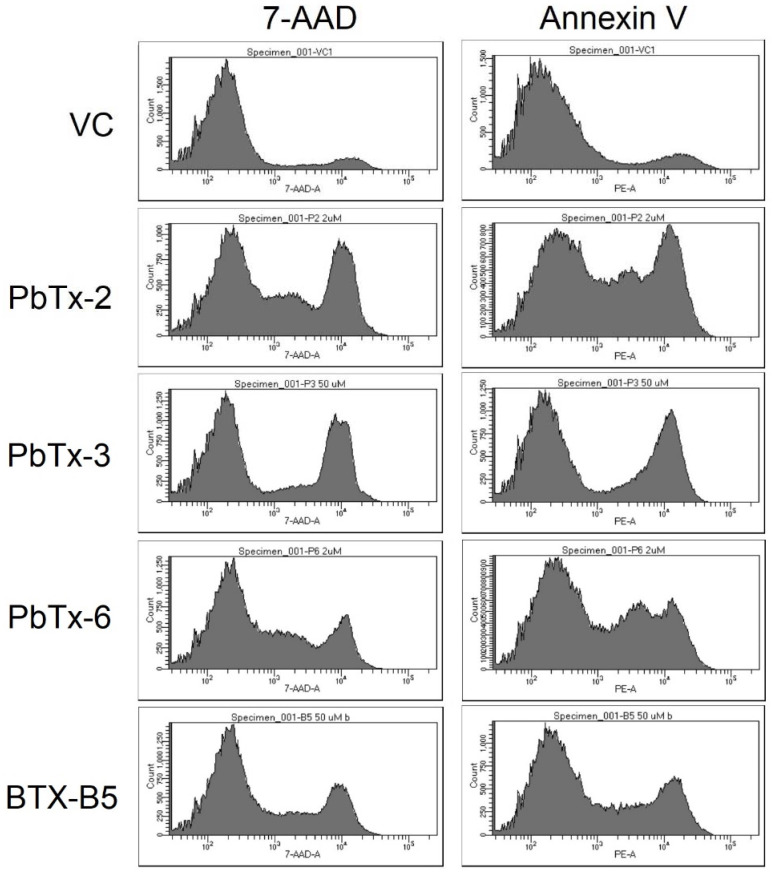
Representative histograms of flow cytometry analysis for the various brevetoxins vs. vehicle control (VC). The left column shows 7-AAD staining, whereas the right shows annexin V.

Additionally, the pattern of staining, and thus annexin V and 7-AAD expression varied among the toxins. The aldehyde-based toxins exhibited a middle peak between the negative and positive populations (Figure 7). PbTx-3 had a steep valley between the negative and positive populations, whereas BTX-B5 landed somewhere in the middle. This pattern can also be seen in the flow cytometry mountain plots of Figure 6. 

### 2.5. Apoptosis and Necrosis from Brevetoxin-Treated PBMCs

**Figure 8:**
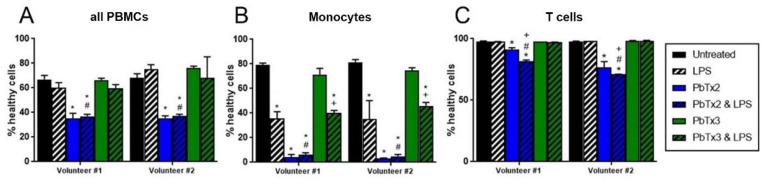
Viability of PBMCs when treated with brevetoxins and/or LPS. PBMCs were isolated from two individual volunteers and treated with either PbTx-2 or PbTx-3 (1 µM), with or without 100 ng/mL LPS. Panel **A** shows all PBMCs in aggregate, whereas Panel **B** is gated to the population of monocytes and Panel **C** is gated to the population of T cells, as previously determined by CD14+ and CD3+ targeting. Black indicates untreated cells, blue PbTx-2, and green PbTx-3, with added stripes for LPS co-treatment. Data is presented as mean ± standard deviation (*n* = 2), with * indicating statistically significant difference from untreated (black), # from LPS-alone (white/black stripes), and + from the corresponding toxin-only treatment (*p* < 0.05).

To confirm the effects seen with the monocyte cell line THP-1 hold true for primary cells, peripheral blood mononuclear cells (PBMCs) were isolated from healthy volunteers and exposed to brevetoxin analogs, and populations of T cells and monocytes were gated for specific cell type analysis. As shown in Figure 8A, the percentage of healthy cells in the aggregate PBMC group (all cells together) was significantly lower than control when treated with 1 µM of PbTx-2, regardless of LPS co-treatment. When cells were gated to only monocytes, there was even further loss in the percentage of healthy cells when treated with PbTx-2 (Figure 8B). Cells co-treated with PbTx-3 and LPS were lower than PbTx-3 alone, but not when compared to LPS alone, indicating that the loss in viability was due to LPS, not PbTx-3 (Figure 8B). When cells were gated to only T cells, there was still loss from PbTx-2, but not to the same extent as for monocytes (14% loss T cells vs. 95% loss monocytes). LPS compounded the loss in healthy T cells, with a significantly lower percentage of healthy cells co-treated with PbTx-2 and LPS as compared to PbTx-2 alone, LPS alone, or VC (Figure 8C). LPS alone or with PbTx-3 co-treatment did not have the same effect on T cell viability as it did on monocytes (Figure 8C vs. Figure 8B). 

**Figure 9:**
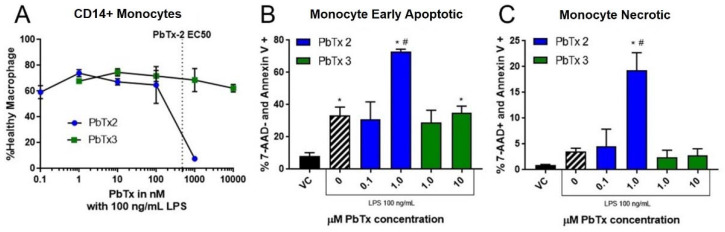
CD14+ monocytes undergo apoptosis and necrosis when exposed to PbTx-2. PBMCs were isolated and treated with either PbTx-2 (100 pM, 1 nM, 10 nM, 100 nM, 1 µM) or PbTx-3 (1 nM, 10 nM, 100 nM, 1 µM, and 10 µM) and 100 ng/mL of LPS. Panel **A** shows the proportion of healthy cells in dose response for both. Panel **B** shows cells that were positive for annexin V but not 7-AAD (i.e., early apopototic), and panel **C** shows cells that were positive for both annexin V and 7-AAD (i.e., necrotic). Data is presented as mean ± standard deviation, with * indicating statistically significant difference from vehicle control (VC), # from VC with LPS (*p* < 0.05).

A dose-response test was then run on one set of PBMCs gated to the monocyte population to examine the effects, specifically of the most toxic doses on primary monocytes. As shown in Figure 9A, PbTx-2 exhibited a dose-response toxicity curve, with an EC_50_ value of 478 nM. PbTx-3 did not elicit a significant decrease in the percentage of healthy cells up to 10 µM, the highest dose tested (Figure 9A). There were significantly more monocytes undergoing both apoptosis (Figure 9B) and necrosis (Figure 9C) with 1 µM PbTx-2. 

## 3. Discussion

Brevetoxins are neurotoxins produced by marine algae that have interesting potential in neuroregeneration following brain injury (e.g., after stroke) due to their ability to increase dendritic arborization and synapse density and to improve motor recovery [6]. The mechanism behind these effects is presumed to be the binding to and persistent activation of VGSCs in neuronal cell membranes. As such, those brevetoxins that bind well to VGSCs have been considered equal in stroke therapy research, particularly PbTx-2 and PbTx-3. However, our findings demonstrate that this should not be presumed because of additional consequences of aldehyde-containing family members.

Our results indicate that PbTx-3 bound similarly to the VGSC receptor as PbTx-2, with a roughly three-fold higher EC_50_, but was much less toxic to THP-1 monocytes, with an approximately 18-fold higher EC_50_. While comparing EC_50_s among assays can be problematic because they measure different things, one would assume that the binding trend (PbTx-2 > PbTx-3 > BTX-B5 > PbTx-6) would hold true for toxicity if toxicity was correlated to VGSC binding as the current paradigm states. In our findings, two sets of clusters arose: a toxicity cluster (PbTx-2/PbTx-6 vs. PbTx-3/BTX-B5) and a VGSC binding cluster (PbTx-2/PbTx-3 vs. PbTx-6/BTX-CBA), which were not aligned. The toxicity cluster held true for monocyte activation states, whereby the aldehyde-containing brevetoxins increase activation states in general, not necessarily polarized to either classical or alternative activation mechanisms. However, the aldehyde-containing brevetoxins do not appear to prime monocytes to respond to external pathogen-associated molecular patterns, such as LPS, because they do not increase the cell-surface expression of the key receptor TLR4. LPS is a component of gram-negative bacterial cell walls and is a potent inflammatory activator, binding to TLR4 on the cell surface. While cell surface expression of TLR4 did not change, this does not eliminate the possibility of changes in total protein expression (e.g., TLR4 inside the cell) or inhibition of the signaling pathway once activated. 

Our results indicate that another mechanism, separate from VGSC binding, is initiating cytotoxicity and activation markers in THP-1 monocytes. This observation is most evident when examining the similarity in effects of PbTx-2 and PbTx-6, as PbTx-2 binds well but PbTx-6 does not, which is assumed to be because the epoxide on the H-ring of PbTx-6 does not allow for the backbone to sit well in the VGSC binding pocket. Indeed, several studies have found inflammatory effects of brevetoxins that may involve sodium channel-independent mechanisms; however, exact alternative mechanisms have yet to be clarified (as reviewed in [30]). We cannot exclude any effect on VGSC activation on the effects we present, especially those where PbTx-3 elicited responses (e.g., LDH release). Indeed, activated monocytes have been shown to increase the expression of VGSCs, particularly in the late endosomes and phagolysosomes, and VGSC activation results in increased phagocytic activity in infiltrating monocytes/macrophages and microglia [31,32]. As such, the observed effects of brevetoxins on monocytes are likely multifaceted and complex. However, it is clear that aldehyde-containing brevetoxins, such as PbTx-2, can have unintended adverse effects on monocytes that would be undesirable during stroke therapy. 

One potential mechanism by which aldehyde-containing brevetoxins elicit cytotoxicity and activation responses from monocytes could be through inhibition of thioredoxin reductase-1 (TrxR) enzyme. Thioredoxin (Trx) mediates the redox state of a cell and is maintained in a reduced state by the TrxR enzymes [33]. Studies have found that PbTx-2 inhibited the Trx/TrxR activity, but PbTx-3 had no effect [34]. PbTx-2 has an α, β-unsaturated aldehyde that forms a weak and disfavored covalent adduct with the C-terminal selenocysteine, resulting in the inhibition of hydrogen peroxide reduction and Trx activity. PbTx-3 showed no similar inhibition [34,35]. However, not all activity of the TrxR was inhibited by PbTx-2, as TrxR was still able to reduce DTNB (or Ellman’s reagent), as the N-terminal redox center of TrxR was unaffected [34]. The Trx/TrxR system is known for being essential in antioxidant defense, but has also been shown to regulate TLR4-dependent responses in immune cells [36,37]. Macrophages treated with Trx-1 were found to downregulate inflammatory cytokine secretion and suppressed LPS-induced differentiation to the M1 phenotype [38]. Another study found that exogenous TrxR downregulated LPS-induced inflammation in macrophages by inhibiting the TLR4-NFkB pathway [39]. Given these findings, one would expect that PbTx-2 binding to and inhibition of Trx/TrxR activity could have wide ranging effects, not only for redox states but also for LPS/TLR4 responses and classical vs. alternative activation of macrophages [37,38]. While exact mechanisms of PbTx-2 interactions with TrxR are still under investigation, our results support the shift in the paradigm that brevetoxin toxicity is not mediated by VGSCs alone.

The cytotoxicity we report herein is likely a combination of VGSC binding and an alternative mechanism that is mediated by the aldehyde functional group (e.g., Trx/TrxR inhibition), and in the case of BTX-B5, the carboxylic acid functional group. The patterns from flow cytometry experiments indicate that PbTx-3 exhibits a relatively simple cell death pattern (mainly necrosis) at high doses, which is likely due to VGSC activation. However, brevetoxins with other reactive groups, including aldehydes and even carboxylic acids, may mediate different mechanisms of cytotoxicity in monocytes, perhaps through enzymatic interactions. One study found that the induction of apoptosis in cancer cells by exogenous electrophiles was dependent on TrxR C-terminal modification [39]. If the aldehydes of PbTx-2 and PbTx-6 act as exogenous electrophiles as others have suggested [34,35], then that would explain how these cells are also able to induce apoptosis in monocytes. Studies are ongoing in our lab to discover the precise mechanisms of action of each toxin and the interactions of VGSC activation along with enzymatic interactions.

Taken together, our results indicate that multiple mechanisms are likely ongoing to reduce monocyte viability, and that primary monocytes react to PbTx-2 similarly to the THP-1 monocyte cell line. These multiple mechanisms of action and interactions therein should be considered when choosing a brevetoxin as a potential therapeutic to avoid off-target effects. As such, brevetoxins that activate VGSCs but do not contain aldehydes (e.g., PbTx-3) should be considered as an alternative in neuroregeneration and stroke therapy studies.

## 4. Materials and Methods

### 4.1. Brevetoxin Extraction and Purification

**Figure 10:**
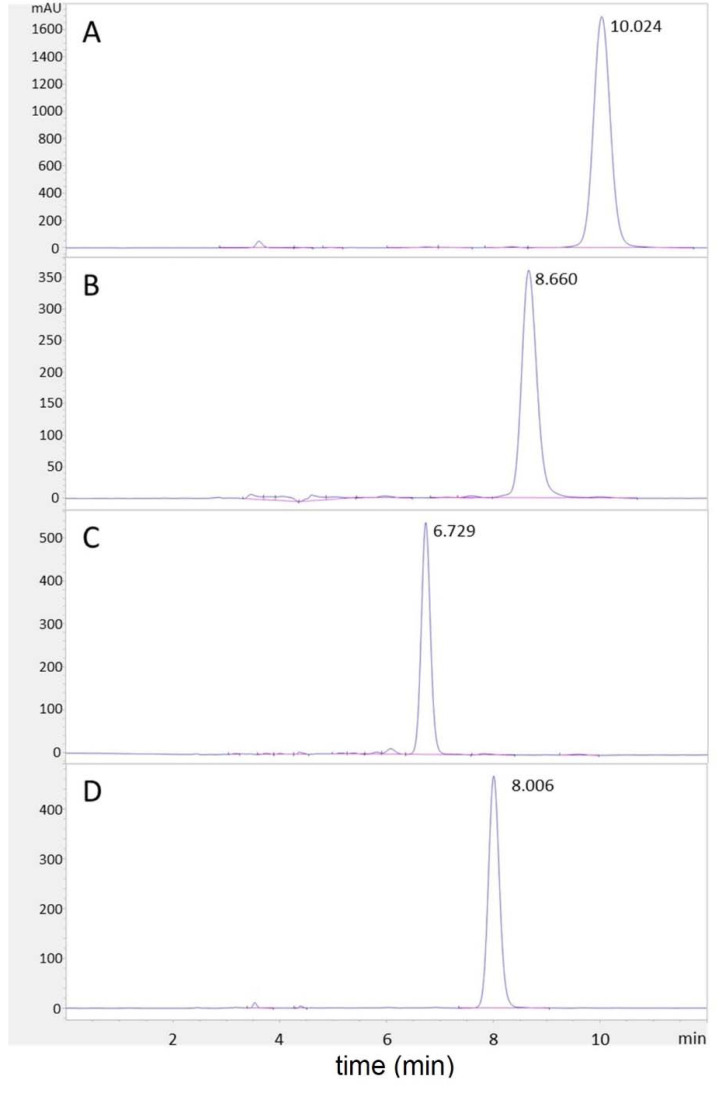
Purity tests of brevetoxins. HPLC-UV chromatograms for purity tests of (**A**) PbTx-2, (**B**) PbTx-3, (**C**) PbTx-6, and (**D**) BTX-B5 analyzed at 215 nm.

PbTx-2, PbTx-3, PbTx-6, and BTX-B5 (PbTx-CBA) were provided by Ms. Susan Niven (UNC Wilmington), using methods similar to what was previously described [25,40,41,42]. Briefly, unialgal cultures of Karenia brevis (Wilson strain) were extracted through liquid:liquid extraction using ethyl acetate. Cultures were homogenized using an IKA ultra turrex and allowed to stand until the layers separated. The organic layer was removed, filtered, and dried under a vacuum. The polyethers were then separated from lipophilic pigments using petroleum ether partitioning with a 50:40:10 petroleum ether:methanol:water mixture. The methanol layer containing the target compounds was then separated using flash chromatography. The fractions containing the PbTxs were identified with TLC and further purified using HPLC. The purity of each brevetoxin was evaluated on an Agilent 1260 Infinity II HPLC equipped with a Zorbax Eclipse Plus C18 Analytical, 4.6 × 250 mm, 5 μm column. The isocratic mode with a mobile phase of 89% methanol and 11% water with 0.1% formic acid, and a flow rate of 0.8 mL/min was utilized for separation. The total run time was 12 minutes, and peaks were detected at 215 nm. Based on peak area integration only, the purities of PbTx-2, PbTx-3, PbTx-6, and BTX-B5 were equal to or greater than 98%, 92%, 94%, and 98%, respectively (Figure 10).

### 4.2. Brevetoxin Receptor Binding Assay

The brevetoxin receptor binding assay was conducted as previously described with slight modifications [19] and using a kit from SeaTox Research, Inc. (Wilmington, NC, USA). Briefly, toxin standards were suspended in methanol to 1 mM and used to generate five more dilutions for 7-point log curves, including methanol blanks. On ice, toxins were combined with chilled receptor preparations that had been lyophilized and resuspended. These reactions were allowed to incubate for 30 minutes before adding the fluorescent ligand for competition and effectively diluting the toxins 100-fold. Assays were incubated on ice and protected from light for an additional 60 minutes. Using a Spectramax iD3 microplate reader equipped with Softmax Pro v7.0.3 (Molecular Devices, San Jose, CA, USA), a background fluorescence scan on each Pall Acroprep Advance filter plate (Pall Corporation, Port Washington, NY, USA) was conducted to account for well-to-well variation with excitation at 483 nm and emission at 522 nm. Reaction contents were then filtered by vacuum and rinsed through each plate before reading again with the wavelengths described. After the fluorescent background was subtracted, RFU data were divided by the blank value (max fluorescence) and analyzed as a percentage of Total Binding. A non-linear regression analysis of % total binding values with Graphpad Prism v9.3.0 (Graphpad Software, San Diego, CA, USA) was used to generate graphics and calculate EC_50_ values. Results are presented as averages plus or minus the standard deviation. EC_50_ data were compared using a one-way ANOVA with Tukey’s multiple comparisons tests and *p* values < 0.05 were considered significant.

### 4.3. Primary PBMC and THP-1 Cell-Line Culture 

The human THP-1 monocyte cell line (ATCC TIB-202) was cultured in RPMI-1640 media (Corning, Corning, NY, USA) with 10% heat inactivated FBS (R&D Systems, Minneapolis, MN, USA), an antibiotic-antimycotic (Gibco, Waltham, MA, USA), and 0.05 mM of 2-mercaptoethanol (Gibco). Primary peripheral blood mononuclear cells (PBMCs) were isolated from healthy volunteers. Briefly, 20 mL of blood was drawn, diluted 1:1 with sterile phosphate buffered saline (PBS) and centrifuged using Lymphoprep density media and SepMate isolation tubes (Stemcell Technologies, Vancouver, BC, USA). The isolated PBMCs were then washed with PBS with 2% inactivated FBS, centrifuged and frozen at −80 °C in RPMI-1640 (Corning) and 10% DMSO. All consent and procedures were approved by the UNCW Institutional Review Board, protocol #21-0168.

### 4.4. Cytotoxicity Assays

THP-1 cells at 7 × 10^5^ cells/mL were plated 100 µL per well in a 96 well plate. Cells were rested in cell culture incubator for two hours, then treated with brevetoxins. The treated cells were incubated for 24 h. An XTT cell proliferation assay (kit from ATCC, Manassas, VA, USA) was performed according to instructions from the manufacturer. An activated-XTT solution was added (50 µL) to each well and the intensity of color was measured following three hours of development with absorbance at 475 nm on the Flex Station III microplate reader (Molecular Devices, San Jose, CA, USA) using the SoftMax Pro 5.2 software (Molecular Devices, San Jose, CA, USA). A reference reading was taken at 660 nm to assess non-specific absorbance, and media background was also subtracted. The percentage of maximum curves were analyzed with non-linear regression curve-fit analysis by GraphPad Prism v7.05 (Graphpad Software, San Diego, CA, USA) to yield EC_50_ values.

A lactate dehydrogenase (LDH) cytotoxicity assay (kit from Invitrogen, Waltham, MA, USA) was performed according to kit instructions. Cells were seeded at 7 × 10^5^ cells/mL, 100 µL per well in a 96 well plate (Greiner, Monroe, NC, USA). Cells rested in cell culture incubator and were then treated with brevetoxins. The treated cells were incubated for 24 h. 50 µL of sample medium was transferred to a new 96 well plate, then 50 µL of reaction mixture was added to each well and incubated for 30 min. Stop solution was added to each well and absorbance was measured at 490 nm and 680 nm (background). Percent cytotoxicity was calculated via kit instructions. Data was analyzed using [agonist] vs. response, variable slope curves, yielding EC_50_ data by GraphPad Prism v7.05. 

For the apoptosis assay, THP-1 cells (ATCC, Manassas, VA, USA) were plated 7 × 10^5^ cells/mL in Greiner 12 well plates (Greiner Bio-One, Monroe, NC, USA) and incubated for 2 h. Cells were then treated with brevetoxins, PbTx-2 and PbTx-6 at 1 µM and 2 µM and PbTx-3 and BTX-B5 at 25 µM and 50 µM. Doses correlate to approximate EC_50_ values and half EC_50_ values for each cluster. The treated cells were then incubated for 24 h and harvested with cold PBS. Fc receptors were blocked for 15 min using goat serum (Gibco, Waltham, MA, USA). Cells were stained using BD Biosciences PE Annexin V Apoptosis Kit (Franklin Lakes, NJ, USA) to kit instructions. Primary gating was performed by positive PE or positive 7AAD, with secondary gating performed by setting up quadrant along positive stain lines when 7AAD was plotted against PE. 

For PBMCs, cells were woken up by removing DMSO cryopreservation media and plated in a 24 well plate (Greiner Bio-One, Monroe, NC, USA) at a density of 194,300 cells/mL. Cells were rested in an incubator for two hours, then treated with brevetoxin for 24 h. Cells were harvested with cold PBS and stained using CD14, then with a PE Annexin Apoptosis Detection kit (BD Biosciences, Franklin Lakes, NJ, USA). Primary gating was by CD14+, followed by quadrant gating of PE versus 7-AAD to determine apoptosis versus necrosis. Both the THP-1 cell line and PBMC were run on BD Celesta flow cytometer. Data was analyzed with one-way ANOVA using GraphPad Prism v7.05. 

### 4.5. Flow Cytometry Assessment for Toll-like Receptor 4 (TLR4), Mannose Receptor (CD206), Interleukin 4 Receptor (IL4Ra), and CD80 Receptor Expression

THP-1 cells (ATCC, Manassas, VA, USA) were plated 7 × 10^5^ cells/mL, 1 mL per well in 12 well plates (Greiner Bio-One, Monroe, NC, USA). Cells were incubated for 2 h, then treated with 1 µM brevetoxins. The treated cells were incubated for 24 h, then harvested with cold PBS. Fc receptors were blocked using 75 µL of goat serum (Gibco, Waltham, MA, USA) then stained with the following fluorescent antibodies: Toll-Like Receptor 4 (TLR4) mouse anti-human conjugated to BV421, Mannose Receptor (CD206) mouse anti-human conjugated to PE-CF594, Interleukin 4 Receptor (IL4Ra) mouse anti-human conjugated with BV510, and CD80 mouse anti-human conjugated with APC-H7, all from BD Biosciences (BD Biosciences, Franklin Lakes, NJ, USA). Stains were applied for two hours on ice in the dark, then rinsed twice with PBS and run on a BD Biosciences Celesta flow cytometer. Cells were gated using the slope of FSC-A versus FSC-H and the results are from the targeted population. The statistical significance was determined with one-way ANOVA using GraphPad Prism v7.05 (Graphpad Software, San Diego, CA, USA). 

### 4.6. Quantification of Cytokines by Enzyme Linked Immunosorbant Assays (ELISAs)

THP-1 cells were seeded in 12-well plates, treated with brevetoxins (1 μM), and incubated at 37 °C for 24 h. The supernatant was collected and stored at −20 °C until ELISAs were performed to measure TNF, IL-6, and IL-1β cytokine production. DuoSet^®^ ELISA kits (R&D Systems, Inc.; Minneapolis, MN, USA) were used to detect cytokines according to the manufacturer’s directions. Absorbance was read at 450 nm using a FlexStation III plate reader (Molecular Devices, LLC; San Jose, CA, USA).

## 5. Conclusions

To date, the primary cause of brevetoxin-mediated effects and toxicity has been assumed to be the activation of the VGSC through binding to site 5. Due to the neuroregenerative properties of brevetoxin’s exposure to neurons, brevetoxins have been proposed as a possible treatment for stroke. However, PbTx-2 and PbTx-3 have been used interchangeably as “brevetoxins” in studies on neuroregeneration. Compounding the issue are the potent inflammatory effects observed from animals and humans exposed to naturally occurring brevetoxins from the environment. In this study, we demonstrated that different analogs of brevetoxins have vastly different effects on monocytes, and those effects are not directly aligned with VGSC binding. Our results demonstrate a paradigm shift in how brevetoxins should be considered as toxins and as drugs. Given the nearly equivalent VGSC binding yet approximately 20-fold (or less) difference in monocyte activation and toxicity, PbTx-3 should be considered for future studies into stroke therapy. 

## Figures and Tables

**Table 1:** EC_50_ values from curves generated from the receptor binding assay (RBA) and XTT cytotoxicity assay (mean ± SD).

Brevetoxin	RBA EC_50_	XTT EC_50_
PbTx-2	9.5 ± 0.74 nM	2.63 ± 0.9511 µM
PbTx-3	27.6 ± 7.56 nM	47.01 ± 1.068 µM
PbTx-6	584.5 ± 129 nM	2.09 ± 1.183 µM
BTX-B5	427.9 ± 123 nM	57.87 ± 0.4809 µM
